# IPNV Antigen Uptake and Distribution in Atlantic Salmon Following Oral Administration

**DOI:** 10.3390/v7052507

**Published:** 2015-05-21

**Authors:** Lihan Chen, Øystein Evensen, Stephen Mutoloki

**Affiliations:** Department of Basic Sciences and Aquatic Medicine, Norwegian University of Life Sciences, Faculty of Veterinary Medicine and Biosciences, P.O. Box 8146 Dep., Oslo 0033, Norway; E-Mails: lihan.h.chen@gmail.com (L.C.); oystein.evensen@nmbu.no (Ø.E.)

**Keywords:** infectious pancreatic necrosis virus (IPNV), Atlantic salmon, oral, anal, uptake

## Abstract

One impediment to the successful oral vaccination in fish is the hostile stomach environment that antigens must cross. Furthermore, uptake of antigens from the gut to systemic distribution is required for induction of systemic immunity, the dynamics of which are poorly understood. In the present study, groups of Atlantic salmon parr were intubated with live or inactivated infectious pancreatic necrosis virus (IPNV), either orally or anally. At 1, 24 and 72 h post infection (p.i.), the fish were sacrificed. Serum was used for assessing IPNV by ELISA, while formalin-fixed head-kidney, spleen, liver and intestine tissues were used for the demonstration of antigens by immunohistochemistry. Both live and inactivated IPNV antigens were observed in enterocytes of the intestines and in immune cells of the head-kidneys and spleens of all groups. In the liver, no antigens were observed in any of the groups. Significantly higher serum antigen OD values (*p* < 0.04) were observed in orally- compared to anally-intubated fish. By contrast, no difference (*p* = 0.05) was observed in tissue antigens between these groups by immunohistochemistry. No significant difference (*p =* 0.05) in serum antigens was observed between groups intubated with live and inactivated IPNV, while in tissues, significantly more antigens (*p <* 0.03) were observe in the latter compared to the former. These findings demonstrate that both live and inactivated IPNV are taken up by enterocytes in the intestines of Atlantic salmon, likely by receptor-mediated mechanisms. Higher IPNV uptake by the oral compared to anal route suggests that both the anterior and posterior intestines are important for the uptake of the virus and that IPNV is resistant to gastric degradation of the Atlantic salmon stomach.

## 1. Introduction

Oral vaccines are the most desirable preparations for use in the aquaculture industry for several reasons: they are stress-free, can be mass-applied to fish of any size, and are not labor intensive [1,2,3]. Despite these advantages, only a few commercial preparations are available on the market at the moment, including those against infectious pancreatic necrosis virus (IPNV), Spring viremia carp virus (SVCV), infectious salmon anemia virus (ISAV), and *Piscirickettsia salmonis* [4,5]. There is no documentation of the protective effects of commercial oral vaccines, although the general understanding is that this is equivocal. At experimental level though, protection has been claimed [6,7]. This status quo highlights the market potential for oral vaccines in the aquaculture industry, but also reflects the challenges faced in their development.

One of the problems associated with oral vaccination of fish is the poor induction of local and systemic immunity by the vaccines. Indeed, oral vaccines come third after injection and immersion preparations in terms of efficacy [8]. Previous studies suggest that this is a result of (a) antigen destruction from exposure to gastric acids and digestive enzymes in the gut of some species of fish; (b) poor uptake of antigens over the intestinal epithelium; and (c) induction of tolerance following oral administration [9,10]. Therefore, to resolve some of these obstacles, several encapsulation formulations with the ability to protect the antigens through the hostile environment of the stomach have been developed, such as alginate beads or microspheres [8,11,12,13]. Nevertheless, even with these formulations, variable results in the vaccination of fish have been reported with different antigen preparations [11,14]. It is also noteworthy that the assessment in these studies were done mainly by examining mortality or survival of fish following challenge (summarized in [8]), whilst antigen uptake remains poorly understood. In the present study, we examined the uptake and distribution of IPNV at early time in selected organs following oral and anal intubation. This has not been well documented previously. Novoa and coworkers attempted to show the uptake and sequential distribution of IPNV in turbot following intraperitoneal injection and immersion infection and drew conclusions at tissue level, but failed to document which cells take up the virus at the portals of entry [15].

Infectious pancreatic necrosis is a disease caused by IPNV and affects salmonids, especially fry at start feeding, parr during fresh water, and post-smolts a few weeks after seawater transfer. IPNV uptake in fish has, in general, not been studied in detail since the 1990s. Available literature shows that the second gut segment is important for uptake of proteins following both oral and anal administration [14,16,17]. For IPNV, however, Sundh and colleagues found that both proximal and distal intestines were routes of uptake in Atlantic salmon [18]. In carp, HRP (solid phase) is taken up by the receptor mediated route and is sorted into the endolysosomal compartment and intercellular spaces [9]. Ferritin and LPS (fluid phase), on the other hand, are taken up through the large supranuclear vacuoles and cannot be observed in intracellular spaces [14,16,17]. How IPNV is taken up is yet unknown and this was the focus of the present study. Specifically, we investigated sequentially the up-take of IPNV from the intestinal lumen and its subsequent distribution to lymphoid organs or to the liver of Atlantic salmon. Our findings suggest that IPNV is taken up in the intestines by enterocytes.

## 2. Results

At 1 h following intubation with live virus, 2/5 fish in the anally intubated group and 3/5 fish in the orally intubated group died prematurely and were excluded from analysis (Table 1). Therefore, the numbers of samples collected at this time point were reduced accordingly. All five fish from each group were sampled from the rest of the time points.

### 2.1. Higher IPNV Antigens Were Detected in Orally-Compared to Anally-Intubated Fish by ELISA

**Figure 1:**
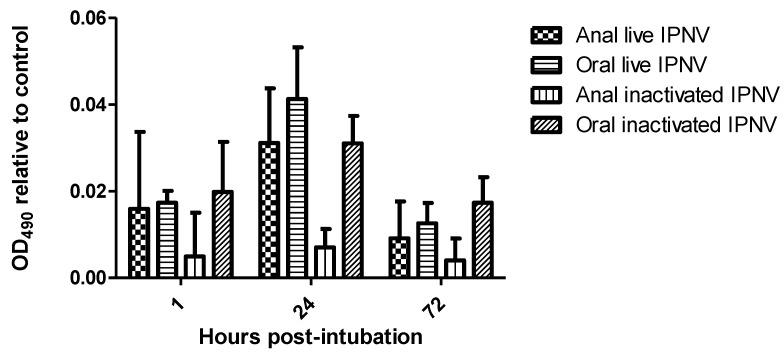
Infectious pancreatic necrosis virus in the serum of different groups of Atlantic salmon at different time points following intubation (using ELISA).

No antigens were present in the control fish. The OD values for treatment exhibited three trends: (1) an increase in IPNV antigens in the serum of all groups from 1 to 24 h post intubation (h.p.i.) followed by a decrease; (2) higher serum antigens (*p <* 0.04) in groups intubated orally (live and inactivated) compared to those intubated anally (Figure 1); and (3) a higher general trend of serum antigens in live groups compared to inactivated ones, albeit non-significantly. Another notable contrast was in the group intubated anally with inactivated IPNV whose OD values were consistently low at all-time points, being comparable to the negative controls (*p =* 0.05).

### 2.2. Detection of IPNV Antigens in Different Tissues by Immunohistochemistry

Table 1 below shows the number of fish in which positive antigens were demonstrated by immunohistochemistry in different groups.

The number of fish per group in which antigens were demonstrated is shown in Table 1. No antigens were detected from the anterior intestine of any of the groups. In contrast, antigens were observed in the posterior intestine of some of the fish intubated both orally and anally with inactivated IPNV from 1 h post intubation (p.i.) onwards.

**Table 1:** Number of fish with positive staining for infectious pancreatic necrosis virus antigens in different tissues following intubation.

Tissue	Time	Live Virus		Inactivated Virus	
	(Hours p.i.)	Anal Intubation	Oral Intubation	Anal Intubation	Oral Intubation
**Posterior intestine**	1	0 *	0 **	1	1
	24	0	0	0	0
	72	0	0	1	1
**Head kidney**	1	3 *	0 **	2	3
	24	0	1	2	0
	72	0	3	3	0
**Spleen**	1	2 *	0 **	1	2
	24	0	0	3	1
	72	0	0	2	0

* *n =* 3; ** *n =* 2 otherwise *n =* 5.

**Figure 2:**
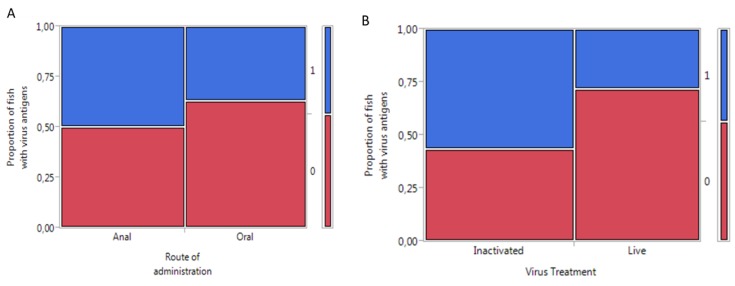
Mosaic plot showing the response of Atlantic salmon to administration of infectious pancreatic necrosis virus: (**A**) comparison between routes of administration and (**B**) comparison between virus treatments. Key: 0 = no antigens detected; 1 = antigen detected in the fish; oral: *n =* 27; anal: *n =* 28; live virus: *n =* 25; inactivated virus: *n =*30.

Significantly more antigens (*p <* 0.03) were observed in fish intubated with inactivated virus compared to live virus when all tissues in each group were summed up (Figure 2).

**Figure 3:**
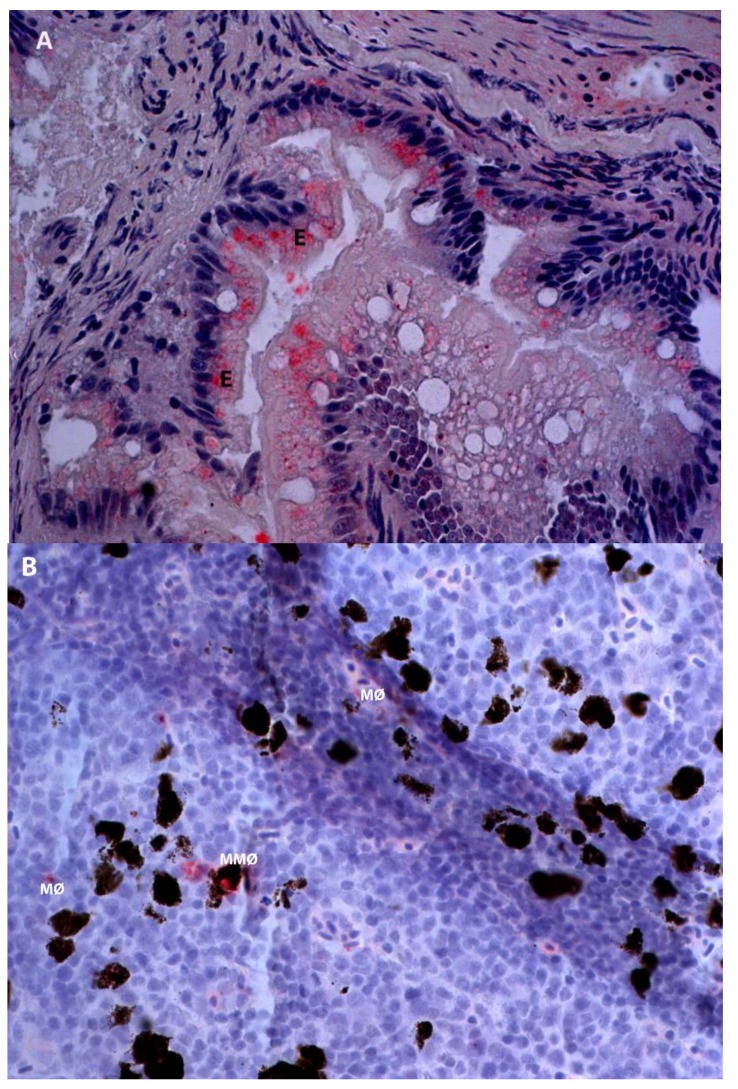
Infectious pancreatic necrosis virus antigens (red stain) in different tissues of Atlantic salmon at designated time points following oral or anal intubation (immunohistochemistry). (**A**) Posterior intestine, inactivated IPNV at 72 h post oral intubation. (**B**) Head kidney, inactivated IPNV at 72 h post anal intubation. Key: E = enterocytes; MØ = macrophages; MMØ = melanomacrophages. Magnification: 40×.

Antigens were observed in the posterior intestine of the inactivated virus groups (both anally and orally) at 1 and 72 h.p.i. The antigens were located in the cytoplasm of enterocytes and macrophage-like cells (Figure 3a). In immune organs (head-kidney and spleen), more antigens were observed in fish intubated with inactivated antigens compared to those with live IPNV. The antigens were observed at all-time points and were localized in macrophage-like and melanomacrophage-like cells (Figure 3b). It is noteworthy that the pattern of positivity was mostly reproduced in several organs, such as the spleen and head-kidney of the same fish although this was not a general rule.

No IPNV antigens were observed in the livers of any of the fish groups.

Surprisingly, no antigens were observed at 24 and 72 h.p.i. post intubation, especially with live virus (Table 1), while for inactivated virus, antigens were demonstrated from several fish.

## 3. Discussion

In the present study, the uptake of IPNV by enterocytes in the posterior intestine, the hematogenous distribution and localization in head-kidney and spleen of Atlantic salmon were demonstrated. Both live and inactivated IPNV antigens were observed in the cytoplasm of enterocytes and macrophage-like cells as early as 1 h.p.i. The antigens were also observed in the named organs at 72 h.p.i. To our knowledge, this is the first report to document the uptake of this virus, both live and inactivated, from the lumen of the intestines. The anticipation is that the mechanisms involved are similar to that of HRP or ferritin, as reported by others [9]. These findings support previous reports that the intestine is important for absorption of macromolecules in fish [17]. Although no antigens were demonstrated in the anterior intestine in the present study, the higher serum antigens in orally intubated groups compared to their anal counterparts, as assessed by ELISA, suggest that both the anterior and posterior intestines as well as the foregut may be important in the uptake of IPNV, as suggested by some [13,17], while contrasting others [9].

Orally administered antigens are believed to be depleted by the time they get to the posterior intestine due to the negative actions of the stomach environment [9,17]. In the present study, anal intubation of antigens was included to contrast the oral in order to test this effect. More antigens were observed in fish intubated orally compared to anally (*p <* 0.04) by ELISA in contrast to the findings of others [9,17]. These results suggest that IPNV is resistant to the low pH and digestive enzymes found in Atlantic salmon stomach. This is hardly surprising given that IPNV is well known to resist chemical and even thermal treatments [19,20,21]. Surprisingly, no antigens were demonstrated in groups of fish anally intubated with live virus at 24 and 72 h.p.i., in contrast to those that were intubated orally. We speculate that there are two reasons for this: firstly, the high threshold of the immunohistochemistry technique making it impossible to detect minute viral quantities (this is contrasted by the ELISA method where antigens were detected, albeit not in a linear relationship); and secondly, the difference in the way antigens intubated anally are handled (since distribution is via venous blood) as briefly discussed below.

The uptake of live IPNV was, in general, comparable to that of the inactivated virus administered orally as measured either by ELISA or immunohistochemistry. This is despite the fact that the live virus has the capacity to multiply and increase in the fish within the time frame of this study. These findings are consistent with reports of others [22,23] and suggest that formalin inactivation of IPNV does not significantly alter its surface structure, thereby allowing the virus to be taken up as efficiently. When it comes to anally-administered inactivated IPNV, however, it is noteworthy that the serum antigens were low at all-time points. In this study, live virus appeared to be more associated with oral route, while inactivated appeared to be more associated with the anal route. The small number of fish, however, precludes firm inferences but this should be a subject of future studies.

In the present study, antigens of both live and inactivated virus intubated either orally or anally were observed in the head-kidney and spleen of the fish from 1 to 72 h.p.i. Antigens were localized in the cytoplasm of macrophage-like cells as well as melanomacrophages. The presence of antigens in these cells is in line with previous reports of antigen retention in immune organs [24]. The head-kidney and spleen of fish are antigen-trapping organs that filter out systemic antigens with melanomacrophage centers serving as focal repositories and may be primitive analogues of germinal centers of lymph nodes [25,26]. Melanomacrophage centers contain lymphocytes and are probably sites where immune activation of trapped antigens occurs [27].

No IPNV antigens were observed in the liver of any groups in the present study. These findings are in agreement with our previous work [28], but contrast the report of others [15]. The reason for this difference is likely methodological as immunohistochemistry was used in this study, while virus re-isolation from cellular fractions was used in the latter. Furthermore, the fish species and probably virus strains were also different. Since salmonid liver, unlike the spleen and kidney, receives mostly venous blood from the gut [29,30,31] and plays a role in the digestion and removal of toxins from the blood, one would expect that all antigens taken up by the intestine would be observed in this organ. The negative result therefore suggests that hepatocytes might not be readily susceptible to IPNV and this view agrees well with previous reports that the liver is one of the last organs to be compromised following IPNV infection [28].

## 4. Materials and Methods

This study was approved by the Norwegian Animal Research Authority.

### 4.1. Cell Lines and Viruses

Asian grouper strain K (AGK) cells [32] were used for the propagation of virus in this study. The cells were grown in L-15 medium (Invitrogen) supplemented by 7.5% fetal bovine serum as well as 10% l-glutamine and were incubated at 28 °C. Chinook salmon embryo cells (CHSE-214) were used for titration of the virus and were maintained at 20 °C in the same medium as AGK cells but with 10% FBS. When infected with IPNV, only 1% FBS was used in the media of both cell lines as well as 1 mg/mL of gentamicin. The incubation temperature was then set to 15 °C until full CPE.

### 4.2. Fish and Rearing Conditions

Approximately 90 Atlantic salmon parr, weighing about 25 g each, were procured from Sørsmolt AS in Sannidal, Norway. The fish were healthy and the hatchery from which they were purchased had had no previous records of IPNV outbreaks in the three years prior to the study. The fish were transported to the Norwegian University of Life Sciences/Veterinary Institute shared wet-lab in Oslo by road in oxygenated bags. One week following acclimatization, the fish were treated with formalin (diluted 1:4000 in water) for 30 min against ectoparasites. The fish were then kept for a further week prior to the onset of the experiment. During the entire experiment the water temperature was 12 °C.

### 4.3. Antigen Administration/Infection of the Fish

Infectious pancreatic necrosis virus grown in AGK cells to a titer of 10^9^ TCID_50_/mL as described above was used. Inactivation of the virus was done as follows: 0.5% formalin (w/v) was added to the virus supernatant followed by incubation at room temperature for 48 h with a magnetic stirrer. Formalin was then removed by dialysis against PBS. To test for inactivation and the presence of residual formalin, fresh CHSE cells were incubated with excessive amounts of inactivated virus supernatant. No CPE or cellular toxicity was observed after 7 days.

Prior to treatment of the fish, the feed was withheld for 24 h. Allocation of the fish into 6 groups was done sequentially by dip netting. Prior to intubation, the fish were anaesthetized by using Benzocaine at 10 mg/L of water. The virus was administered into the fish by using a 1 mL syringe and tube. Treatment groups, comprising 15 fish each, were as follows: (1) Live IPNV administered orally; (2) Live IPNV administered anally; (3) Inactivated IPN administered orally; (4) Inactivated IPNV administered anally; (5) L-15 medium only administered orally; and (6) L-15 medium only administered anally. Each fish received 0.3 mL of the preparation. Marking of the fish was by fin-clipping and each of the four groups (Live-oral; Live-anal; inactivated-oral; Inactivated-anal) was kept in a separate tank. The controls (media only) were kept together with the inactivated virus groups.

### 4.4. Sampling

At 1, 24, and 72 h post intubation (h.p.i.), 5 fish from each group were sacrificed and sampled. Samples of blood, liver, spleen, kidney, anterior intestine (immediately caudal to the pyloric caeca) and posterior intestine (1 cm cranial to the anus) were collected. Blood samples were centrifuged on site and then serum was aspirated and transferred to clean tubes for storage at −80 °C until required. The rest of the tissue samples named above were preserved in 10% phosphate buffered formalin.

### 4.5. Enzyme-Linked Immunosorbent Assay (ELISA)

In order to assess the amount of IPNV in the blood of the different groups of fish, 96-well plates were coated with 100 μL serum from experimental fish diluted at 1:40 in coating buffer (0.1 M Carbonate buffer pH 9.6). The plates were then incubated at 4 °C overnight. The next morning, the plates were washed before blocking with 100 μL of 5% dry milk for 2 h at room temperature. Unless otherwise stated, all washing steps were done using 200 μL of PBST/well. Dry milk was diluted with PBST while antibodies were diluted with 1% dry milk. Following blocking, the plates were washed and then incubated with 100 μL of 1:1000 rabbit anti-IPNV antibodies (K95) [33] at room temperature for 1 h. Following a washing step, 100 μL of secondary antibody, peroxidase-labeled goat anti-rabbit (DAKO; Glostrup, Denmark) diluted at 1:1000 dilution, was incubated in each well at room temperature for 1 h. After another washing step, 100 μL OPD substrate was added to each well and incubated at room temperature for 15 min. The reaction was then stopped by adding 50 μL/well 1 M H_2_SO_4_. OD values were detected by using an ELISA reader at 492 nm absorbance.

### 4.6. Immunohistochemistry

Staining of tissues was carried out as described by Evensen and Lorenzen 1996 [34]. Briefly, after de-paraffinization and rehydration, tissue sections were blocked with 5% BSA (Sigma Aldrich, St. Louis, MO, USA) diluted in 1 M Tris buffer solution (TBS) pH 7.6 for 30 min. Subsequently, 150 μL of rabbit anti-IPNV serum (K95) diluted at 1:1000 in 2.5% BSA was added to each slide. After incubating for 30 min at room temperature, the slides were washed. All washing steps were carried out using 1 M Tris-buffer pH 7.6 with 1% Tween 20. Biotinylated goat anti-rabbit antibody (DAKO; Glostrup, Denmark) was then added for 30 minutes. After washing, streptavidin alkaline phosphatase (Sigma-Aldrich) was added and incubated for 30 min. Following washing, fast red substrate (Sigma-Aldrich) was added to each slide and incubated for 10 min. The reaction was stopped by immersion of slides in running tap water for 5 min. Counterstaining was carried out using Hematoxylin dye for 2 min and then washing in tap water. After mounting with glycerol, the slides were observed under a light microscope.

### 4.7. Statistical Analysis

Differences in antigen scores as detected by ELISA between oral *versus* anal intubation; and between live *versus* inactivated virus groups were analyzed by Analysis of Variance (ANOVA) on cumulative data that was normalized by square-root transformation. To analyze differences between treatment types (live *versus* inactivated) and routes of intubation (oral *versus* anal) on the response (antigen present/not present as detected by immunohistochemistry), Fisher’s exact test was used with the help of the JMP^®^ statistical software (SAS Institute Inc., Cary, NC, USA).
